# Fraudulent Participation in Online Qualitative Studies: Practical Recommendations on an Emerging Phenomenon

**DOI:** 10.1177/10497323241288181

**Published:** 2024-11-16

**Authors:** Khaylen Mistry, Sophie Merrick, Melissa Cabecinha, Susanna Daniels, John Ragan, Miran Epstein, Louisa Lever, Zoe C. Venables, Nick J. Levell

**Affiliations:** 1 Department of Dermatology, Norfolk and Norwich University Hospitals Foundation Trust, Norwich, UK; 2 Norwich Medical School, 6106 University of East Anglia, Norwich, UK; 3 MRC Clinical Trials Unit at UCL, Institute of Clinical Trials and Methodology, 4919 UCL, London, UK; 4 Research Department of Primary Care and Population Health, Institute of Epidemiology and Healthcare, 4919 UCL, London, UK; 5 Melanoma Focus, Cambridge, UK; 6 Patient and Public Representative, Norwich, UK; 7 4617 Queen Mary University of London, London, UK; 8 Legal Services, 6107 Norfolk and Norwich University Hospitals Foundation Trust, Norwich, UK

**Keywords:** fraudulent participation; scammers; monetary incentives; online recruitment; research ethics

## Abstract

Fraudulent participation is defined in the following as participation in research by individuals who, for one reason or another, intentionally provide false responses. Qualitative studies are at an increased risk of fraudulent participation when online recruitment and participation are used, and monetary incentives offered. Fraudulent participation threatens data quality and subsequent evidence-based practice, yet validated guidance on how to tackle it is lacking. This paper offers a critical reflection thereon by three separate qualitative research groups that experienced fraudulent participation in collaboration with a patient representative, a bioethicist, a legal expert, a journal deputy editor, and a chief executive of a national charity. The Prevent FRaudulent Online STudy participation (P-FROST) recommendations provide advice on (1) Study set-up (including team members and study design), (2) Monetary incentives and recruitment, (3) Data collection (screening and interview considerations), and (4) Analysis, reporting, and support. The reflection which balances the diverse perspectives of patients, researchers, funders, ethics boards, and legal teams puts forward the P-FROST recommendations to identify and prevent fraudulent participation throughout the design, ethical approval, and implementation of online qualitative research.

## Introduction

The COVID-19 pandemic has acted as a catalyst, accelerating the transition of qualitative and quantitative data collection including surveys, interviews, and focus groups from face-to-face/in-person to online formats (Roberts et al., 2021). The use of online modalities in this context is likely to persist, as they offer numerous benefits; however, they are not devoid of drawbacks (Table 1) (Sah et al., 2020). Recent publications have highlighted fraudulent participation as an emerging issue for qualitative studies using online recruitment and participation and monetary incentives (Lawlor et al., 2021; Roehl & Harland, 2022; Sefcik et al., 2023; Woolfall, 2023). Fraudulent participants are individuals who intentionally provide false responses/data, for their own personal benefit, in this case likely for the financial gain (Chandler et al., 2020). The frequency of these fraudulent events is unknown though likely under-reported. This threat to data quality, evidence-based practice, policy, and research funds is poorly understood, and validated guidance is lacking.

**Table 1.** Advantages of Online Versus Face-to-Face Qualitative Data Collection.

Advantages of online qualitative data collection (Irani, 2019; Lo Iacono et al., 2016; Seitz, 2016)	Disadvantages of online qualitative data collection (Irani, 2019; Lo Iacono et al., 2016; Seitz, 2016)
• Access to wider patient population and larger sample size, including broader geographical reach and access to vulnerable or difficult to access groups who may not engage with healthcare services, have physical or psychological disabilities, or belong to minority ethnic backgrounds, enabling adequate recruitment. • Time and cost-efficient, allowing participants and researchers to flexibly schedule meetings despite busy work and personal lives, collect more data, and conduct more studies. • Participants are more relaxed because they are in a comfortable and familiar environment of their choice and thus may provide less socially desirable responses to sensitive questions. • Environmentally friendly as reduced travel emissions to attend interviews.	• More challenging to develop effective rapport. • Difficult to pick up on non-verbal cues as often cannot observe full range of body language from waist below. • Restricted participant cohort to those with access and skill in using required technology, although 92% of UK adults were frequent users of the internet in 2020 (Office of National Statistics, 2021). • Technical issues such as internet connection or sound quality. • Risk of fraudulent participation. • Can be difficult to provide support and express compassion over video call.

Three separate qualitative research groups that experienced fraudulent participation collaborated with a patient representative from a cancer support group, a bioethicist, a legal expert, a journal deputy editor, and a chief executive of a national cancer charity involved in two of the studies, to critically reflect on three cases of fraudulent participation. The aim of this work was to raise awareness of this growing issue and carefully balance the diverse perspectives of patients, researchers, funders, ethics boards, and legal teams to put forward practical recommendations on how to identify, prevent, and tackle fraudulent participation in online qualitative research. These recommendations may serve qualitative researchers, peer reviewers, and ethics boards when designing, implementing, and appraising online qualitative studies.

## Examples of Fraudulent Participation

### Case Study 1: UK Skin Disease Online Interview Study

To identify patients’ future research priorities, participants with skin cancer were recruited by skin cancer charities using websites and social media. A Patient and Public Involvement and Engagement (PPIE) group of seven participants expressed interest in the study within a few days. Emails received were from “Gmail” accounts with a common name followed by numbers, for example, john.doe112@gmail.com. After expressing interest, all participants were notified of a £50 digital voucher reimbursement. Pre-interview questionnaires were completed but some key information was left blank, and participants all claimed to be from a similar minority demographic background, incongruous but not inconceivable for the condition of interest. Individual interviews were conducted and recorded on Zoom. None of the seven participants turned their cameras on during interviews, audio quality was poor, some answers were vague or contradicted information that had been previously provided in their pre-interview questionnaires, and responses were almost identical among the seven participants. Although the voucher company and other study participants confirmed the digital voucher codes were functioning and had been used, the seven participants claimed the codes did not work in an attempt to be paid twice. This was followed by a high volume of emails to the research team and hospital complaints department, some threatening to make critical statements on social media and take legal action unless urgent requests to provide another code were met immediately. Those participants who claimed the codes did not work were reassured the issue was being investigated and the study was paused. A legal and fraud specialist team provided advice to researchers not to communicate further, and events were reported to the police through the Action Fraud website (https://www.actionfraud.police.uk/). Following discussion with the sponsor, funder, and a patient representative, the research group successfully submitted amendments to the ethics committee to remove the monetary reimbursement and make video recording a mandatory component of interviews. Any further participants about whom the team had concerns were discussed collaboratively among the research team. Retrospectively, the fraudulent cases were easily identified with strong similarities and clues to distinguish them from genuine cases. Fraudulent cases were excluded from the analysis.

### Case Study 2: Interviews to Discuss Awareness and Knowledge of HIV Pre-Exposure Prophylaxis (PrEP) Among Women in England

This study was open to women, age 18 and over, who resided in England at the time of participation. Interviews could be conducted via telephone or Microsoft Teams, depending on participant preference. The study was advertised via the project Twitter (now X) account and through paid advertisements on Meta social media platforms. Participants were offered a £20 voucher reimbursement, redeemable at several retailers in the United Kingdom, for participating in an hour-long interview. Interested parties were invited to complete a pre-interview questionnaire, which included questions on the potential participant’s demographics (to facilitate purposive sampling) and other items to confirm the respondent’s eligibility to participate. The research team then reviewed the pre-interview questionnaires and contacted respondents to provide further information on the study and to schedule interviews.

Throughout the recruitment period, the pre-interview questionnaire received numerous suspicious submissions. For example, several submissions were made in quick succession with different contact details, but similar or identical answers to the other questionnaire items. In some cases, the answers did not meet the study eligibility criteria (e.g., the respondent identified as male), whereas in others nonsensical answers were provided (e.g., the respondent listed their city of residence as “crumpets”). In these instances, the provided contact details were used to thank respondents for their interest in the study and inform them that they were not eligible to take part. However, it was not always possible to identify ineligible or fraudulent participants from the pre-interview questionnaire responses, so additional measures were taken to ensure that only genuinely eligible participants were interviewed.

Due to the sensitive nature of the interviews, it was important to balance each participant’s comfort and interview preferences with ensuring only eligible participants took part. During the interviews, sensitive topics such as a participant’s sexual behavior and experiences were discussed; therefore, participants had the option to keep their webcams off when online interviews were recorded. Before starting each interview, participants were asked to confirm their answers from the pre-interview questionnaire. When a participant’s answers were not consistent with the responses collected in the questionnaire, the participant was thanked for their interest in taking part and informed that they were not eligible to participate in the study.

### Case Study 3: Online Focus Group Study to Understand the Perspectives of Patients With Cancer on Clinical Trial Designs

This focus group study aimed to understand the perspectives of patients with advanced cancer regarding potential clinical trial designs. The study advertised for participants through cancer charities including support groups, patient forums, and social media platforms. Initially, the study accrued participants at the expected rate. The advertisement was then placed on a charity social media platform, and this was the first occasion where details of the £25 voucher reimbursement were included. Following this advertisement, a rapid flurry of applications was received within hours. All these applicants registered with “Gmail” accounts. During the first focus group, most of the spurious participants kept their cameras and microphones off and did not meaningfully engage in the discussion. Furthermore, information given during the introductions was inconsistent with the information provided in the background questionnaire. Many participants appeared preoccupied with receiving the voucher. Concerns were raised by both facilitators and some authentic participants that not everyone taking part was genuine or eligible for the study. Following the first focus group, all consent forms and background questionnaires were reviewed. There was a suspicious pattern of registration for many participants. This included many applicants from the same demographic background, which was atypical for the disease. In addition, some details in the background questionnaire were implausible. An approved ethical amendment required all participants to have a pre-screening video call with a researcher prior to taking part in a focus group. It was also highlighted in the participant information sheet (PIS) that a camera and microphone would be required to take part. The implementation of the screening video call was successful with all subsequent focus groups including only eligible participants who were fully engaged in the discussion. The participants from the first focus group who did not meaningfully engage were excluded from the analysis.

## Identifying Fraudulent Participation

Identifying fraudulent participants may be difficult, and researchers should adopt a structured approach to build and interpret evidence in the context of their study when substantiating fraudulent participation (Ridge et al., 2023). Information during recruitment, data collection, and analysis should be reviewed in an iterative process to enable early detection of fraudulent participation. Table 2 describes the collection of red flags identified across the qualitative studies in which the authors were involved. The accumulation of red flags can alert researchers to the possibility of fraudulent participation warranting team discussion on the management of this issue; however, some flags in isolation may be more suspicious than others. Previous studies have set cut-offs for participant inclusion based on the number of red flags identified (Salinas, 2023).

**Table 2.** Red Flags to Raise Suspicion of Fraudulent Participation in Qualitative Research.

Red Flag	Example
Recruitment	
Participant email address Generic private email addresses often composed of a common name (perhaps a celebrity’s name) and series of numbers. Fraudulent participants may use the same email platform or duplicate addresses.	John.Doe999@...mail.com
Participant expression of interest in study A short generic email response expressing interest in study. No mention of specific details of the study, how they found out about the study, or why the participant wants to be involved. This response could be copied and pasted across different studies. May include text which has been copied from the recruitment advertisement verbatim. Omits any personal details beyond their first name. May have poor grammar and a blank subject line. An influx of these suspicious responses within a short time period should raise suspicion. Emails may be sent at unusual times of the day which could suggest that the applicant is in a different time zone.	“I am interested in your research.” “Hello, With great delight I am pleased to be enlightened more about this study and will love to receive a PIS that helps explains this study widely.i am interested and will like to be reached to take part in the study.”
Data collection from screening questions, interviews, and focus groups	
Screening questionnaires Unexpected information on demographics or disease. Free text answers may be left blank, be vague, or contain inappropriate responses. There may be patterns to answering Likert scale questions (tend not to select extreme options) (Salinas, 2023). Metadata may show responses were completed in an unrealistically fast time.	Invalid postcode Multiple applicants with unusual age or ethnicity for study disease.
Interview/focus group audio and video Preference for online rather than telephone interviews or focus groups. Camera turned off during interview or focus group. Poor audio quality. Organized fraudulent participants may all have similar accents or use the same phraseology.	Zoom interview where participants chose to keep camera off or stated camera not working. Accent and muffled audio made it difficult to understand the participant.
Participant answers during interview/focus group Vague or brief answers to questions. Unable or reluctant to elaborate on answers when probed. Incongruent responses compared to pre-screening questionnaire. Unable to clarify on these discrepancies on inquisition. Duplicate or similar responses among fraudulent participants.	Mentioned they were treated at a private hospital in London but were not able to provide the specific name of the hospital. Stated they had stage 1 cancer on the pre-interview questionnaire, but in the interview the participant stated they had stage 4 cancer that had spread to multiple organs.
Participant rapport Difficult to build rapport. The participant seems distracted during interview. Shorter interview duration (Roehl & Harland, 2022).	Short one-word answers Shuffling paper in background
Data analysis and reimbursement	
Analysis Unexpected themes generated	Themes were not in keeping with expert consensus or current literature.
Voucher reimbursement Participants get in touch soon after the interview to enquire about timing and method of payment. These emails may be identical or similar among fraudulent participants. Reluctance to provide an address to send physical vouchers or provide bank details for Bankers’ Automated Clearing System (BACS) payments.	Requested online digital Amazon vouchers that can be used internationally

## Recommendations to Prevent FRaudulent Online STudy Participation (P-FROST)

**Figure 1.**
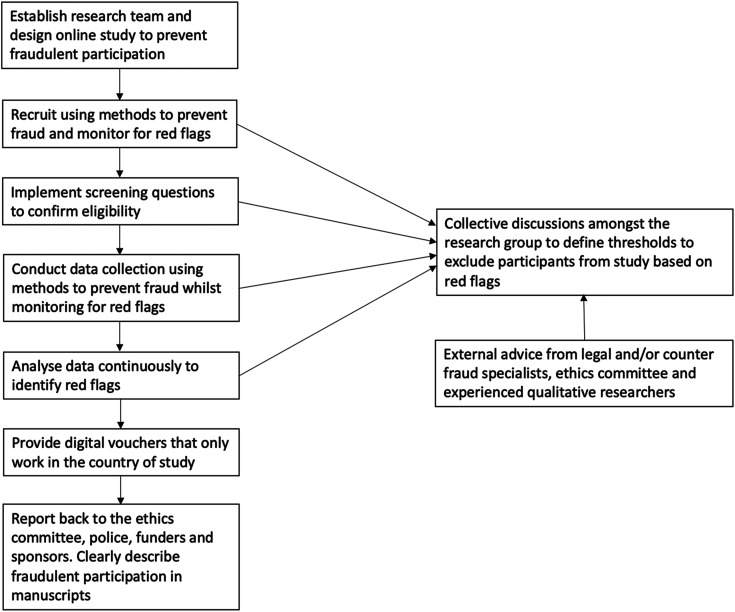
Example of a standardized approach to preventing, identifying, and managing fraudulent participation using P-FROST.

Recommendations to P-FROST were informed by the above case studies and following several multidisciplinary research team discussions among co-authors who inputted their respective expertise and experiences of methods to prevent fraudulent participation. All co-authors contributed to and agreed to the final set of recommendations. A literature review was completed to identify relevant literature that contained recommendations. PubMed, Embase, CINAHL, Web of Science, Scopus, and PsycINFO were searched on 13 May 2024 using the key terms “fraudulent participation” and “qualitative.” Study titles and abstracts were searched, and MeSH terms were used when possible. Grey literature was identified by reviewing the first 1000 hits from Google Scholar following the search “Fraudulent participation in qualitative research.” The references of any identified review articles were screened for further relevant citations. The search terms were chosen to efficiently identify papers and limit the number of hits to those most relevant. A summary of the P-FROST recommendations to prevent fraudulent participation in online qualitative research is provided in Table 3. Figure 1 illustrates how P-FROST can be implemented within an online qualitative study.

**Table 3.** Summary of Prevent FRaudulent Online STudy Participation (P-FROST) Recommendations.

Study Aspect	Recommendation
Study set-up	
Research team—the research team should share the necessary skills to identify and tackle fraudulent participation (Sefcik et al., 2023).	• Involve experienced qualitative researchers to collect and analyze data to enable early identification of fraudulent participation. • Consult computer experts to provide technical solutions to provide optimal online conditions for genuine participants and enable effective identification and prevention of fraudulent participation (Teitcher et al., 2015). • Ensure all research team members have completed relevant information security training delivering awareness of the latest scams and methods to recognize phishing. • If fraudulent participation is suspected, seek legal, police, and/or specialist counter fraud advice early depending on the severity and scale.
Study design—methods to identify and prevent fraudulent participation should be integrated into the study design of online qualitative research. These methods should be decided carefully and ethically approved prior to commencing the study.	• Patient and public involvement (PPI) should be utilized to ascertain whether planned interventions to protect against fraud would be agreeable or limit participation. • Pilot studies could be conducted to offer insight into aspects that do not work well so that modifications can be made.
Monetary incentives and recruitment	
Monetary incentives—a modest incentive may be appropriate to compensate participant’s time; however, disproportionate incentives may amount to undue influence (Ambuehl et al., 2015). Not providing monetary incentives may exclude specific groups such as working professionals or those with childcare commitments. It remains unclear whether monetary reimbursement is becoming more common for research projects and high quality up-to-date data is lacking (Fry et al., 2005). The National Institute for Health and Care Research (NIHR) provides guidance for the United Kingdom on when incentives are appropriate and how much should be offered; however, what is appropriate in one culture may be excessive or inadequate in others (Research) (National Institute for Health and Care Research, 2024).	• Researchers should carefully consider whether or not to advertise monetary incentives during initial recruitment. Advertising incentives throughout initial recruitment may encourage fraudulent participation with subsequent compromise of research integrity. Conversely, not advertising monetary incentives may compromise recruitment. If the research group decides that concerns regarding research integrity take precedence over concerns of inadequate recruitment, then only following a participant’s expression of interest inform them of any reimbursements using the PIS. The method of reimbursement should be detailed in the PIS. • Preferred methods of reimbursement to mitigate fraudulent participation include Bankers’ Automated Clearing System (BACS); however, bank payments can be challenging to set up leading to delays in payment. They may also result in issues related to data protection or privacy and be unworkable in practice when recruiting populations reluctant to divulge personal information. A document recording details of payment method and date can be helpful to track payments (Jones et al., 2021). • Posting vouchers ensures only one voucher per address and confirms the participant’s geography; however, this is vulnerable to participants claiming they have not received the voucher (which may be difficult to disprove), participants may be opposed to sharing their address, and it incurs financial and time overheads. • Where digital vouchers are employed, they should be restricted to those that only work in the country where eligible participants live, for example, “Love2Shop^©^” vouchers for the United Kingdom. • To enable researchers to withhold payments, a paragraph could be included in the PIS and consent form describing circumstances where reimbursement may not be sent, for example, until valid confirmation of a participant’s identity is received.
Recruitment methods—recruitment methods should be carefully decided to best reach the target population while mitigating the risk of fraud.	• Where possible, recruitment either in person or through closed groups for the population of interest such as the healthcare centers, charities, and support groups may be less susceptible to fraudulent participation (Salinas, 2023). Caution should be adopted when recruiting by snowball sampling or openly accessible sources such as open social media or trial recruitment websites (Sefcik et al., 2023). Researchers should be astute to the aforementioned red flags during recruitment. • Research teams may benefit from the incorporation of Internet Protocol (IP) address tracking within recruitment strategies. Studies which recruit from a defined geographic region may decide to examine IP addresses to confirm a participant’s location. Some software providers enable IP address review; however, this may consume researcher time and subscription costs. Multiple participants using the same IP address could suggest fraudulent activity; however, different participants that are using a public computer (e.g., library, hospital, and university) may have the same IP address. Sophisticated fraudulent participants may employ technologies such as a virtual private network (VPN) to hide their IP addresses (Kramer et al., 2014). To support the appropriate implementation of these technologies, universities should have dedicated information security teams with expertise in this area to consult with when planning and conducting online studies. Participants must also be informed of their use.
Data collection	
Screening questions—screening questions are often used to identify and confirm eligibility during participant recruitment and support purposive sampling prior to data collection. These questions can also help identify, deter, and prevent fraudulent participation.	• Screening questions to confirm participant eligibility could include demographic or disease-specific information (e.g., subtype or severity) and management (names and dates of key prescriptions or surgery). Researchers may consider requesting that participants provide verification documents (e.g., Healthcare Identification Number) to confirm they meet study eligibility criteria. It is imperative that the PIS and consent form explain what identifiable information will be collected and how it will be used, clearly stating that the information used to confirm eligibility will not be reported as part of the study findings. • Repeated or similar screening questions can help confirm consistency in answers provided (Simone, 2019). • Free text screening questions can precipitate nonsensical answers and prevent participants moving through the questionnaire quickly (Pequegnat et al., 2007). • Researchers may ask participants to confirm their responses to screening questions before commencing the interview or focus group. A pre-interview video call allows researchers to review information provided in screening questionnaires to improve data quality and confirm eligibility. It also gives the opportunity to resolve technical issues, address participant queries, and establish rapport between the researcher and participant. • Researchers should consider the minimum information required in screening questions to verify eligibility while protecting against identifiable information in line with data protection and confidentiality laws. Researchers should avoid enquiring beyond this to avoid participant fatigue and maintain anonymity; asking participants to provide identifiable information may decrease trust in the research team (Lawlor et al., 2021). Fraudulent participants may pass screening through the provision of falsified information or being eligible to take part and subsequently taking part multiple times under different identities. Highlighting eligibility criteria on study advertisements is important for participants to be able to self-identify and self-select for the study but may make it easier for fraudulent participants to pass screening.
Interviews/focus groups—interviews and focus groups should be designed with the primary aim of obtaining rich and meaningful data with awareness of approaches to flag potentially fraudulent participants.	• A requirement that cameras must be turned on during interviews or focus groups may deter fraudulent participants while offering additional benefits in building rapport and yielding key body language information to guide the interview or focus group (Jenner & Myers, 2019). In focus groups, participants may feel more comfortable contributing if they can see other participants (de Souza et al., 2024). However, it may deter participation from people who would prefer not to have their camera on. Requiring participants to turn their video on even if just momentarily prior to video recording could discourage fraudulent participants (Ridge et al., 2023). • Researchers may request to see a form of identification prior to commencing the recording. Such requirements should be embedded within the consent form and PIS. • Semi-structured interview guides could be designed in a manner that would potentially reveal fraudulent participation. Open questions can gauge participants’ depth of interest and knowledge on the topic. Vague, nonsensical, or duplicate answers should be probed for detail and congruency (Jones et al., 2021).
Analysis, reporting, and support	
Data analysis—a structured and collaborative approach to data analysis enables both high-quality data synthesis and early identification of fraud.	• Wherever possible, data should be analyzed as it is collected. • A code “suspicious response” could be utilized, and excerpts later discussed. • A reflexive journal provides an audit trail of researcher observations and thoughts which yield rich insight on such issues. • Regular meetings should be scheduled to discuss whether interim findings match those expected by experts and existing literature. Principal investigators should encourage colleagues to voice uncertainty regarding authenticity. Debriefing on suspected fraudulent participants can ameliorate issues of self-criticism (Ridge et al., 2023). Regular meetings and debriefings are pertinent if numerous researchers are conducting and analyzing interviews to facilitate timely identification of issues.
Managing data from fraudulent participants—following detailed discussions, the research team should arrive at a consensus on whether to exclude data from potentially fraudulent participants.	• Seeking advice from experienced qualitative researchers and literature review may guide judgement. • The approach to these decisions should be systematic and consistent. Establishing a conceptual framework to define thresholds for exclusion may encourage a standardized approach to these decisions (Figure 1). • At the earliest point, if concerns arise based on the above red flags, politely apologize to potentially fraudulent participants that the research will not be proceeding further and thank them for their interest. Legal and/or counter fraud specialists can be consulted to help compose this correspondence. • If fraudulent participants have already completed the study, then reimbursements should be honored.
Reporting transparency—it is important to involve the necessary parties following research fraud to obtain the required advice, allow critical appraisal of research, and raise awareness of this growing issue.	• It is recommended that feedback is provided to research ethics committees to raise awareness. • Where appropriate, if a scam operation is suspected, make the police aware of any potential fraud, which in the United Kingdom can be done through the Action Fraud website. • Funders and sponsors should be updated throughout. • Researchers conducting online qualitative studies should detail measures taken to prevent, identify, and manage fraudulent participation in manuscripts. Where fraudulent participation is identified, the proportion excluded and rationale for exclusion should be stated in the results of study publications.
Supporting research teams—being the subject of fraud can have a distressing impact on the research team. Utilization of an emotionally supportive infrastructure should be sought for victims of research fraud.	• Researchers should engage with university and employee support services, alongside regular planned discussions within the research team to ensure there is a structured support system in place to manage the impact of fraudulent participation. A proactive approach is recommended, where the support systems researchers plan to utilize should be highlighted in the research protocol. • The NHS Every Mind Matters (https://www.nhs.uk/every-mind-matters/) and Samaritans (https://www.samaritans.org/how-we-can-help/contact-samaritan/talk-us-phone/) can also assist with the emotional impact that fraud can cause.

## Discussion

Researchers routinely prioritize convenience and anonymity for participants to maximize study accessibility; however, this may predispose online studies to fraud and researchers should balance participant protection against data integrity considering the various perspectives of patients, researchers, funders, ethics boards, and legal teams (Roehl & Harland, 2022). Qualitative researchers should incorporate methods to detect and prevent fraudulent participation into ethics and funding applications.

Qualitative studies often aim to include a diverse range of participants and experiences, and any anti-fraud measures must be carefully considered to ensure they do not impact the recruitment of genuine participants. This is particularly relevant when conducting research with underrepresented, hidden, or other marginalized populations and/or when the research concerns a sensitive or stigmatized subject. For example, IP address tracking or requiring identity checks may discourage participants from signing up, as these may be seen as undermining or compromising the confidentiality and anonymization of the research. This may be a particular issue when the research focuses on sensitive topics such as sexual health and experience, illegal activities such as experience of drug use or sex work, or stigmatized and politicized issues, such as migration status. Additionally, requirements such as participants needing to have their webcams on during online focus groups and interviews may exclude those with poor internet connection or inadequate digital devices from taking part. In such cases, the anti-fraud measures must be weighed carefully against the needs and preferences of potential participants, and strategies that rely on the researcher’s experience and skills (e.g., confirmation of screening questions and pre-interview discussions) may be preferable to strategies that collect additional information or data from participants that are not relevant to the research outputs. Anti-fraud measures may require substantial time and financial commitments to identify and avoid fraud. Researchers should strategically decide on the most efficient and applicable techniques, considering the range of potential threats from fraudulent participation to their study.

Researchers must be mindful of how unconscious biases can influence their decision-making when deciding whether a participant is genuine. In case study 1, presented above, members of the fraudulent cohort were from a minority demographic that is underrepresented in skin cancer research, and initially the research team was keen to have their experiences included in the study. When issues with this cohort arose, the researchers were concerned that their suspicions of fraud could, in part, be due to unconscious bias or a lack of awareness of issues that minority groups have with research participation. Researchers were concerned that these participants were not familiar with how the research was conducted which could have explained why their answers during interviews were generic or vague. Language barriers may have resulted in miscommunication during interviews. These participants may have experienced digital poverty and therefore were not able to have their cameras on. Ultimately, the accumulation of red flags as described in case study 1 led to the decision that participation was fraudulent, and the participants were excluded from the study. It is important to recognize the range and diversity of experiences from minority groups and not exclude these groups because their experiences do not fit with current understanding of a disease in the general population.

High-quality recommendations and frameworks to address the issue of fraudulent participation have been produced elsewhere (Davies et al., 2024; Lawlor et al., 2021; Roehl & Harland, 2022). The strength of the recommendations in this study stems from the unique combination of authors. A patient representative ensured the recommendations were acceptable and would not deter genuine participants. The underlying rationale of the recommendations was balanced by a bioethicist while delivering insight into the ethical review board’s perspective. A legal expert ensured the recommendations abided by applicable legislation while a journal deputy editor provided input on the necessary information that should be reported by authors on the steps taken to prevent and manage fraudulent participation when preparing a manuscript to enable critical appraisal. Finally, a chief executive of a national charity provided a funders’ viewpoint, especially when making difficult decisions regarding monetary reimbursement. Although not formally validated, the above recommendations were tested in the context of the three unique qualitative case studies and found to be robust and effective in preventing, identifying, and managing fraudulent participation. Recommendations are not a panacea; implementing them alone cannot guarantee the eligibility of all participants. Rising artificial intelligence, social media use, and sophisticated technology make the prevention and identification of fraudulent participation increasingly challenging, and thus recommendations will need to be adaptive and responsive. Artificial intelligence may equip fraudulent participants with capability to quickly and accurately respond to research questions during screening, online surveys, interviews, and focus groups (Májovský et al., 2023). Artificial intelligence detectors tools exist; however, their effectiveness to detect the authenticity of content requires further validation (Májovský et al., 2023). Recruitment through social media platforms has become increasingly accepted, yet studies may be liable to opportunistic discovery by fraudulent participants who create false unverified accounts to follow research accounts who advertise recruitment for studies offering monetary reimbursement. These recommendations can only protect against the known approaches that fraudulent respondents take to enter online qualitative research. Research groups should reflect on their confidence in the methods used to identify fraudulent participation. Decisions on exclusion should be considered through an ethical lens, where a genuine participant is removed, their input has been supressed from the study, potentially losing key information. Contrarily, retaining fraudulent responses distort the value of legitimate responses and may alter study findings. Participation of fraudulent participants in focus groups can be distressing to genuine volunteers. Further validation studies are required to explore the effectiveness and acceptability of respective recommendations to hinder fraudulent participation and develop robust guidance. Future research should also assess the frequency of fraudulent participation and the reasons for its occurrence.

In conclusion, the described recommendations offer a consistent approach toward identifying and preventing fraudulent participation in online qualitative research. These recommendations contribute toward an evolving conversation to ensure that convenient online qualitative methodology is not at the expense of data quality. Academic directors and institutions should raise awareness of this issue and take steps to educate on fraudulent research participants within curriculums. Emphasis on transparency among researchers is key to ease pressures around participant recruitment and publications which deflect from the need for honest, accurate data. We encourage researchers to use the red flags and recommendations and actively engage with the ongoing discussion regarding this issue through letters, review articles, and further studies. Future partnerships where patients, researchers, funders, and ethics committees work together to establish consensus groups are imperative to establish validated guidance, alert current researchers, and ameliorate this growing problem.
